# Prevalence of poor and rapid metabolizers of drugs metabolized by CYP2B6 in North Indian population residing in Indian national capital territory

**DOI:** 10.1186/2193-1801-1-34

**Published:** 2012-10-16

**Authors:** Ekta Varshney, Nilanjan Saha, Monika Tandon, Vikesh Shrivastava, Shakir Ali

**Affiliations:** 1 Department of Biochemistry, Jamia Hamdard, Hamdard Nagar, New Delhi, 110062 India; 2 Ranbaxy Clinical Pharmacology Unit, Ranbaxy Laboratories Limited, Gurgaon, India

**Keywords:** Cytochrome P450, CYP2B6, Bupropion, Drug metabolism, LCMS/MS, India

## Abstract

Identification of poor and rapid metabolizers for the category of drugs metabolized by cytochrome P450 2B6 (CYP2B6) is important for understanding the differences in clinical responses of drugs metabolized by this enzyme. This study reports the prevalence of poor and rapid metabolizers in North Indian population residing in the National Capital Territory.

The prevalence of poor and rapid metabolizers was determined in the target population for the category of drugs metabolized by CYP2B6 by measuring plasma bupropion, a drug metabolized by CYP2B6, and its metabolite. Bupropion (75 mg) was administered to 107 volunteers, and the drug (bupropion) and its metabolite (hydroxybupropion) were determined simultaneously by LCMS/MS in the plasma. CYP2B6 activity was measured as hydroxybupropion/bupropion ratio, and volunteers were categorized as rapid or poor metabolizers on the basis of cutoff value of log (hydroxybupropion/bupropion). Significant differences were observed between the mean metabolite/drug ratio of rapid metabolizers (Mean = 0.59) and poor metabolizers (Mean = 0.26) with p<0.0001. Results indicate that 20.56% individuals in the target population were poor metabolizers for the category of drugs metabolized by CYP2B6. Cutoff value defined in this study can be used as a tool for evaluating the status of CYP2B6 using bupropion as a probe drug. The baseline information would be clinically useful before administering the drugs metabolized by this isoform.

## Introduction

Human cytochrome P450 2B6 (CYP2B6) is involved in the biotransformation of a variety of clinically important drugs such as the antiretroviral nevirapine (NVP) and efavirenz (EFV), which are used to treat AIDS and/or stop the spread of HIV infection (Erickson et al. 1999; Ward et al. 2003), antimalarial drug artemisinin (Simonsson et al. 2003; Mehlotra et al. 2006) and other drugs including cyclophosphamide, tamoxifen, diazepam, and bupropion (Lang et al. 2001; Wang and Thompkins 2008). However, the rates with which these drugs are metabolized vary considerably in individual hepatic microsomes, and this variation is believed to be caused by CYP2B6 isoforms, besides the environmental factors such as the enzyme inducers. Clinical importance of genetic variations and role of ethnicity of *CYP2D6*, *CYP2C19*, *CYP2C9,* and *CYP2D6* are well known (Adithan et al. 2003; Anitha and Banerjee 2003; Kumar et al. 2010; Lamba et al. 1998ab) but *CYP2B6* has only recently been recognized to code for a highly variable enzyme of potential clinical importance (Lang et al. 2001; Lamba et al. 2003). More than 100 DNA variations have been reported in *CYP2B6* gene, and many of them show extensive linkage disequilibrium giving rise to distinct haplotypes. The spectrum of functional consequences of these variations is wide and includes null alleles with no detectable function and/or expression (alleles CYP2B6*8, *12, *15, *18, *21), alleles with partially reduced function/expression (CYP2B6*5, *6, *7, *11, *14, *19, *20, *21) (Lamba et al. 2003; Klein) and alleles with increased expression (CYP2B6*22) (Zukunft et al. 2005).

Clinical relevance of *CYP2B6* variation has been demonstrated for the anti-HIV drug efavirenz. Common clinical practice of administering the same dose to all patients leads to profound differences in drug plasma concentration, which is correlated with patient genotype (Tsuchiya et al. 2004; Novoa et al. 2005). Patients with high drug concentrations are at risk of developing concentration related central nervous system toxicity, including insomnia, fatigue, and headache, which often lead to discontinuation of therapy. Thus, for a drug such as efavirenz, dose adjustment based on *CYP2B6* genotype could prevent administration of too-high doses, and increase the safety and efficacy of therapy. Further, CYP2B6 variant genotyping at baseline may allow clinicians to identify patients who are at risk of treatment failure or drug toxicity (Novoa et al. 2005; Ramachandran et al. 2009). Some of these variations are rare, but many are common, with allele frequencies between 10% and almost 50%, depending on the population (Klein et al. 2005; Solus et al. 2004). Ethnic or racial inter-individual CYP2B6 polymorphism in various populations has been reported in Caucasians (Lang et al. 2001), Japanese (Hiratsuka et al. 2002and Hiratsuka et al. 2004), African-American-Hispanic (Lamba et al. 2003; Hesse et al. 2004), Korean (Cho et al. 2004), Mongolian (Davalkham et al. 2009), Spain (Novoa et al. 2005), and South Indians (Ramachandran et al. 2009), but not in North Indian population, and, hence, CYP2B6 was selected in this study.

### Aim of the study

This study was aimed at to find out the prevalence of poor and rapid metabolizers for the category of drugs metabolized by CYP2B6 in the target population by measuring plasma bupropion, a drug metabolized by CYP2B6, and its metabolite.

## Method

### Clinical study

Study protocol and corresponding informed consent form (ICF) were reviewed by the Institutional Review Board, and procedures were in accordance with the Helsinki Declaration of 1975, as revised in 2000. Subjects were informed before initiation of the study through an oral presentation regarding the purpose of the study, procedures to be carried out, potential hazards and rights of the subjects. Subjects (170) were selected randomly from the volunteer bank of clinical pharmacology unit of Ranbaxy Laboratories Limited. The volunteer bank comprises of healthy volunteers from the Indian National Capital Region (INCR), which includes the metropolitan area encompassing the entire national capital territory (Delhi) and urban areas of neighboring states of Haryana, Uttar Pradesh and Rajasthan. Subjects were selected on the basis of inclusion and exclusion criteria after obtaining written informed consent. Medical histories and demographic data were recorded. Each subject underwent physical examination and laboratory tests of hematology, hepatic and renal function. Hematological parameters were analyzed on fully automated five part differential count autoanalyzer, Sysmex XT 1800xi, procured from Transasia Co. The biochemical parameters, which included plasma glucose, serum blood urea nitrogen, serum creatinine, serum total bilirubin, serum alkaline phosphatase, serum alanine and aspartate aminotransferases, serum cholesterol, and urine drug of abuse, were analyzed on a fully automated biochemistry analyzer, Dimension Rxl (Seimens Diagnostics, USA), according to manufacturer’s instruction. Urinalysis, routine and microscopic examination, was done by manual dipstick method (Multistick from Seimens Diagnostics). Rejection or selection of subjects was based on specific clinical and medical examination as shown in Table 1. Subjects were kept under medical supervision in the clinical pharmacology unit of Ranbaxy Laboratories Limited, New Delhi. Bupropion (Wellbutrin^R^, GlaxoSmithKline, USA) (75 mg) was administered orally to selected (107) volunteers along with 240 ml of water under the supervision of a trained Medical Officer. The EDTA blood sample (6 ml each) was collected at 0, 1, 3, 6 and 10 h after drug administration. Number of volunteers was calculated statistically based on the prevalence of percentage poor metabolizers present worldwide. Sample size of 100 volunteers was calculated statistically. The prevalence of CYP2B6 in Indian population is ~40%, and therefore, a sample size of 100 subjects was calculated and found sufficient to estimate the prevalence with expected 95% binomial confidence interval ranging 30 to 50%. Based on the simulation with higher sample size (200 or 300), not much benefit was found in precision with a sample size of 100 subjects.

**Table 1:** **Number of volunteers rejected on the basis of medical and clinical examination**

Physical and medical examination	Number of volunteers
ECG	4
Medical Examination	12
Outliers in hemogram and biochemical parameters	11
Drug of Abuse positive	18
Serology positive (HIV, HCV, HbsAg)	6
Others	12
Total (Rejected)	63
Selected for phenotyping (Total - Rejected)	170–63 = 107

*A total number of 170 volunteers were screened and 107 were recruited for the study.*

### Determination of bupropion and its metabolite by LCMS/MS

CYP2B6 activity was determined by calculating hydroxybupropion/bupropion ratio in plasma by LCMC/MS on Waters Quattro premier mass spectrometer. Samples were analyzed using a set of calibration standards spiked in human plasma. Three levels of quality control samples were distributed through each batch of study samples assayed to monitor the performance of testing. Experiments were carried out by liquid-liquid extraction with ethyl acetate selected as an optimum extraction solvent for the estimation of both bupropion and its metabolite. Briefly, 100 μl of 0.5N sodium carbonate was added to 100 μl of plasma and 50 μl of internal standard dilution (5 μg/ml diazepam solution) in a clean test tube. Sodium carbonate was added to the extraction buffer to maintain the drug and metabolite in un-ioned state. The mixture was vortexed for a minute, and 4 ml extraction solution (ethyl acetate) was added to it followed by centrifugation at 4,000 rpm for 5 minutes. The organic layer (3.5 ml) was removed and transferred to a fresh tube and mixed with 50 μl of 0.1N HCl. Supernatant was vortexed for 10 seconds and kept in an evaporator under nitrogen at 50°C for 10 minutes, and then reconstituted in 250 μl diluent consisting of 80 parts of water and 20 parts of acetonitrile. Reconstituted solution was injected into LCMS for bupropion and hydroxy bupropion estimation. Six replicates of aqueous dilutions of bupropion, hydroxybupropion, and diazepam were injected and their peak response ratios were recorded to check the interferences or specificity. The calibration ranges for bupropion (1–500 ng/ml) and hydroxybupropion (5–2500 ng/ml) were selected. Calibration curve was accepted if the back-calculated concentrations of minimum 75% of calibration standard (without including standard zero) were within 85% and 115% of the nominal concentration. Coefficient of correlation of linear regression (r^2^) of calibration curve was 0.98. Six different batches of biological matrix (9204, 122412, 123892, 123852, 123202 and 122501) were analyzed for selectivity exercise. Six blank samples spiked with LLOQ (lower limit of quantification) were processed by sample preparation procedure. Peak area was evaluated at the retention time of analyte and internal standard. Selectivity was accepted only if the peak area in blank at retention time of analyte was <20% of mean peak area of analyte at LLOQ and <5% of mean peak of internal standard in calibration standard for internal standard. Long-term stability of analyte was evaluated using low and high QC samples stored below −50°C in deep freezer for a period of 15 days. Six replicates of low and high quality control samples were used for each stability exercise. The stored QC samples were analyzed against freshly spiked calibration curve.

### Data analysis

The population was categorized as poor and rapid metabolizers for the group of drugs metabolized by CYP2B6 on the basis of CYP2B6 activity. The enzyme activity was determined by evaluating hydroxybupropion/bupropion ratio. The concentration of bupropion and hydroxybupropion was quantified from the blood samples drawn at pre-dose, 1 h post-dose, 3 h post-dose, 6 h post-dose and 10 h post-dose from the subjects kept in-house till 10 h post-dose. Based on the concentration of the parent drug at the above mentioned time points, *t*_*max*_ (time to reach the drug at the highest concentration) of 3 h was selected for the evaluation of CYP2B6 activity. The frequency histogram was constructed with log (metabolite/drug at *t*_*max*_) *versus* the number of volunteers. The presence of different categories of individuals was indicated in frequency histogram if the frequency histogram was different from the normal distribution. On visual inspection of frequency histogram, approximate antimode position was established as the point on graph where two different modes are separated. However the exact antimode was derived by probit plot analysis. This is a graphical method in which standard deviates of a normal distribution are plotted against the log (metabolite/drug). Deviations from linearity in probit plots have been interpreted as existence of polymorphism. Scatter type chart was prepared with log (metabolite/drug) on x-scale and probit on y-scale. Trendlines were added to the plot to get best linear fit. Based on the selected trendline, a polynomial equation of regression was obtained. Intercept at x was the antimode. Individuals having log metabolite/drug ratio less than the antimode were classified as poor metabolizers. The mean of the ratio (metabolite/drug) of poor and rapid metabolizers was analyzed by student *t*-test to evaluate the significance of difference between poor and rapid metabolizers and the P value of less than 0.05 was accepted as statistically significant.

## Results

**Figure 1:**
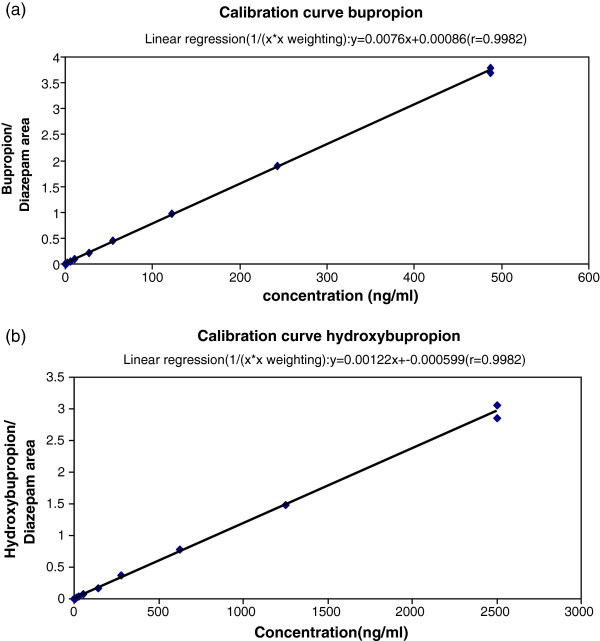
**Calibration curves for the estimation of (a) bupropion, and (b) hydroxybupropion.**

**Figure 2:**
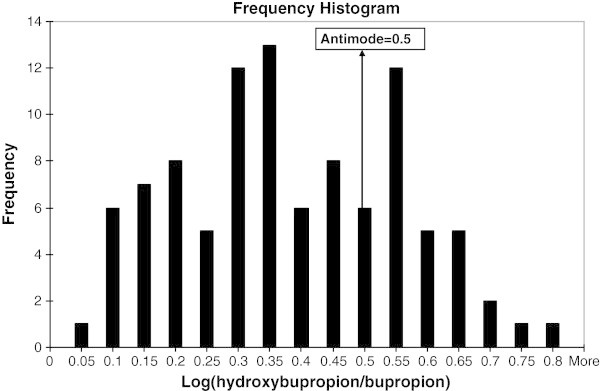
**Frequency histogram plotted as log ratio of hydroxybupropion/bupropion*vs.*number of individuals.** Arrow indicates antimode at 0.5.

**Figure 3:**
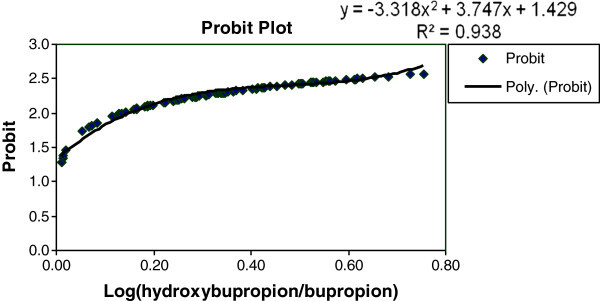
**Probit plot of log (hydroxybupropion/bupropion)*vs.*probit.**

None of the volunteers reported any undesirable effect or adverse event during or after the study. No interference was observed in the retention time of bupropion or hydroxybupropion. The retention time of 1.16 (bupropion), 1.13 (hydroxybupropion), and 1.70 min (diazepam) was tuned. The sensitivity of estimation of bupropion and hydroxybupropion or percent coefficient variation at LLOQ was 6.59% and 6.87%, respectively. The estimation procedure was specific as no interfering peak was observed in six different batches of biological matrix. The coefficient of correlation of linear regression (r) was 0.9982 for bupropion and 0.9982 for hydroxybupropion. Calibration curves of bupropion and hydroxybupropion are shown in Figure 1. Percent accuracy of the calibrators and quality control samples was between 85 and 115%. Precision for bupropion and hydroxybupropion was estimated as mean %CV (coefficient of variation). It was between 3.9 and 5.7% for all three levels of quality control samples. The values were within FDA defined limit of <15%. The frequency histogram and probit plot analysis described the bimodality of the studied population with respect to log (metabolite/drug ratio) (Figures 2 and 3). Regression analysis done on the probit plot yielded a best linear fit at R^2^ = 0.938. The trendline equation (y = −3.318x^2^ + 3.747x + 1.429) was obtained. On solving the equation, intercept at x-axis, which was actually an antimode, was found to be 0.5 [log (hydroxybupropion/bupropion)]. Individuals having log ratio of hydroxybupropion/bupropion <0.5 were categorized as poor metabolizers. Based on the antimode value, 20.56% of population was categorized as poor metabolizer for the category of drugs metabolized by CYP2B6. Significant difference was observed between the mean ratio of metabolite/drug of rapid metabolizers (Mean = 0.59) and poor metabolizers (Mean = 0.26) with P<0.0001 using student *t*-test (Table 2).

**Table 2:** **Evaluation of mean ratio of poor and rapid metabolizers of CYP2B6 using*t*-test**

Variables	Poor metabolizers	Rapid metabolizers
Mean	0.260093	0.590142
Variance	0.022124	0.004614
Hypothesized mean difference	0	-
Degree of freedom	76	-
*t* Statistics	−15.2236	-
P (T<=t) one-tail	4.34E-25	-
*t* Critical one-tail	1.665151	-

## Discussion

This study reports the prevalence of poor and rapid metabolizers for the category of drugs metabolized by CYP2B6 in the target population. Interest in CYP2B6 has been developed by an ever-increasing list of substrates metabolized by this isoform as well as polymorphic and ethnic variations in the expression and activity of CYP2B6. Previous *in vitro* heterologous expression studies have shown that the polymorphism found in alleles CYP2B6*5, *6, *7, and *9 can alter the expression and/or activity of the enzyme (Ariyoshi et al. 2001; Iwasaki et al. 2004; Jinno et al. 2003). The functional significance of CYP2B6 variants has been shown for a variety of drugs. For example, in AIDS clinical studies, CYP2B6 variants have been associated with 2- to 4-fold higher plasma EFV and NVP (Haas et al. 2004; Rotger et al. 2005; Rodriguez-Novoa et al. 2005; Tsuchiya et al. 2004) in HIV patients; ≥2-fold higher plasma EFV concentration is associated with neuropsychological adverse effects (Haas et al. 2004; Rotger et al. 2005; Marzolini et al. 2001; Hasse et al. 2005). Besides the antiretroviral drugs, CYP2B6 variants have also been found to influence the metabolism and pharmacokinetics of bupropion (an antidepressant) (Hesse et al. 2004) and cyclophosphamide (an anticancer and immunosuppressive) (Xie et al. 2003). This study is the first attempt to identify poor and rapid metabolizers of the drugs metabolized by CYP2B6 in north Indian population residing in the national capital. Bupropion is widely used in phenotyping of CYP2B6 (Faucette et al. 2000; Kirchheiner et al. 2003; Rotger et al. 2007; Chung et al. 2011) and has been found to be a safe and tolerable drug. We did not report adverse effect of the drug during the clinical trial, and found it a safe, suitable and tolerable drug. Bupropion and its metabolites were measured in the plasma by LCMS/MS. Validation parameters were within the acceptable limits as recommended in FDA. Analysis of the results based on frequency histogram and probit analysis revealed that 20.56% of the target population was poor metabolizer. The prevalence of poor metabolizers, which we observed in this study, was comparatively lower than West Africa (54%) (Malhotra et al. 2006), Papua New Guinea (63%) (Malhotra et al. 2006), Spain (40%) (Novoa et al. 2005), Mongolian (35.5%) (Davalkham et al. 2009), Japanese (32.6%) (Gatanaga et al. 2007), Han Chinese (32.9%) (Guan et al. 2006), African American (54.6%) (Klein et al. 2005), Ghanians (59.7%) (Klein et al. 2005), Caucasians (38.9%) (Blievernicht et al. 2007) and Koreans (23.9%) (Klein et al. 2005). In India, percentage of poor metabolizers was 40% in South Indian population (Ramachandran et al. 2009). In comparison, North Indian population reported 20.56% poor metabolizers, which is considerably lower. The difference might be attributed to the life style and genetics of these two diverse groups of populations in India. In a study by Rendic (2002), nutrition has been reported to play an important role in drug metabolism and affect some of the CYP isoforms including 1A1, 1A2, 1B1, 2A6, 2B6, 2C8, 2C9, 2C19, 2D6, 3A4 and 3A5. Similarly, occupational exposure to hazardous chemicals is reported to affect CYP1A1 and CYP2E1 (Nan et al. 2001). In this study, we could not evaluate the correlation of phenotype with genotype, which would be advantageous to understand the genetic background of the difference in poor and rapid metabolizers. However, the prevalence of 20.56% of poor phenotype for CYP2B6 reported in this study cannot be ignored because of its involvement in the metabolism of drugs commonly used for the treatment of cancer, HIV infection and depression, where the treatment is usually long term, and these drugs may be toxic due to poor metabolism.

## Conclusion

The antimode or cutoff defined in this study can be used as a tool for evaluating the status of CYP2B6 activity using bupropion as a probe drug. The baseline information would be clinically useful before administering the drugs metabolized by this isoform.
